# Effects of Cholecystokinin in the Supraoptic Nucleus and Paraventricular Nucleus are Negatively Modulated by Leptin in 24-h Fasted Lean Male Rats

**DOI:** 10.1111/j.1365-2826.2010.01982.x

**Published:** 2010-05

**Authors:** C Caquineau, A J Douglas, G Leng

**Affiliations:** Centre for Integrative Physiology, University of EdinburghEdinburgh, UK

**Keywords:** area postrema, Fos, hypothalamus, NTS, oxytocin, pSTAT3, satiety

## Abstract

Cholecystokinin (CCK) and leptin are two important satiety factors that are considered to act in synergy to reduce meal size. Peripheral injection of CCK activates neurones in several hypothalamic nuclei, including the supraoptic (SON) and paraventricular (PVN) nuclei and neurones in the brainstem of fed rats. We investigated whether peripheral leptin would modulate the effects of CCK on neuronal activity in the hypothalamus and brainstem of fasted rats by investigating Fos expression in the PVN, SON, arcuate nucleus, ventromedial hypothalamus (VMH), dorsomedial hypothalamus (DMH), area postrema (AP) and the nucleus tractus solitarii (NTS). Male rats, fasted for 24 h, received either one i.p. injection of vehicle, leptin or CCK-8 alone, or received one injection of vehicle or leptin before an i.p. injection of CCK-8. We found that CCK increased Fos expression in the PVN and SON as well as in the NTS and AP, but had no effect on Fos expression in the arcuate nucleus, VMH or DMH compared to vehicle. Leptin injected alone significantly increased Fos expression in the arcuate nucleus but had no effect on Fos expression in the VMH, DMH, SON, PVN, AP or NTS compared to vehicle. Fos expression was significantly increased in the AP in rats injected with both leptin and CCK compared to rats injected with vehicle and CCK. Unexpectedly, there was significantly less Fos expression in the PVN and SON of fasted rats injected with leptin and CCK than in rats injected with vehicle and CCK, suggesting that leptin attenuated CCK-induced Fos expression in the SON and PVN. However, Fos expression in the NTS was similar in fasted rats injected with vehicle and CCK or with leptin and CCK. Taken together, these results suggest that leptin dampens the effects of CCK on Fos expression in the SON and PVN, independently from NTS pathways, and this may reflect a direct action on magnocellular neurones.

Cholecystokinin (CCK) is an important satiety factor that regulates how much food is consumed during a meal. Released from the gut during eating, CCK acts via CCK-A receptors that are located on the nerve endings of gastric vagal afferents projecting to the nucleus tractus solitarii (NTS) in the caudal brainstem. In rats and mice, systemic injections of CCK induce expression of Fos (the protein product of the immediate-early gene *c-fos*) in neurones in the NTS, and also in several hypothalamic nuclei that receive projections from the NTS, including both parvocellular and magnocellular oxytocin neurones in the paraventricular nucleus (PVN) and supraoptic nucleus (SON) (1). At least some of the parvocellular oxytocin neurones of the PVN that are activated by CCK project back to the NTS, where there is dense expression of oxytocin receptors (2). There, they activate a descending vagal efferent pathway that regulates the passage of food through the gut (3). As a result of the activation of magnocellular oxytocin neurones, CCK stimulates magnocellular oxytocin secretion from the posterior pituitary into the systemic circulation (4). Peripherally-administered oxytocin stimulates sodium excretion (natriuresis) by a combination of direct actions at the kidney and indirect actions of atrial natriuretic peptides from the heart, It has therefore been proposed that oxytocin secreted in response to CCK reflects a reflex pathway that stimulates natriuresis to compensate for the sodium intake accompanying feeding (5). Oxytocin is also released in the hypothalamus from both parvocellular and magnocellular neurones to inhibit food intake by acting on important satiety centres such as the ventromedial nucleus of the hypothalamus (VMH), which expresses oxytocin receptors in abundance (6).

The satiety-inducing effects of CCK appear to be modulated by prevailing concentrations of leptin, a hormone released from adipose tissue that is an important long-term regulator of food intake (7–9). Plasma concentrations of leptin increase with feeding and decrease with fasting and, in rats fasted for 48 h, systemic injections of CCK are less potent at reducing food intake than in fed rats, although this is reversed by leptin administration (8). Leptin receptors are expressed in afferent vagal neurones in the NTS (10–12) and some reports indicate that leptin receptors are particularly abundant in the SON and PVN (13–15). The actions of leptin in the brain are classically mediated by a Janus kinase-signal transducer and activator of transcription (STAT) pathway, resulting in the regulation of target gene expression (16–18). A recent microarray study of the PVN reported that, in fasted mice, in which oxytocin is, as for rats, anorexigenic, oxytocin mRNA expression is reduced, and that this effect of fasting is reversed after systemic administration of leptin, suggesting that leptin may have an important action in modulating the rate of synthesis of oxytocin (19).

In the present study, we tested the hypothesis that leptin potentiates the responsiveness of supraoptic and paraventricular neurones to CCK. We first studied CCK-induced expression of Fos protein in fasted rats, which have low circulating concentrations of leptin, and then studied the effects of pre-treatment with leptin on the CCK response.

## Materials and methods

All animal experiments were performed under project and personal licenses awarded by the UK Home Office, and in accordance with Home Office guidelines.

### Animals

Adult male Sprague-Dawley rats were housed individually under a 12 : 12 h light/dark cycle, at an average room temperature of 22 °C with food and water available *ad lib*. Twenty-four hours before beginning experiments, food was removed for all rats. Rats had free access to drinking water throughout.

### Intraperitoneal injections

Experiments began at 3 h after lights on. In a first experiment, 24-h fasted rats (250–270 g body weight) were injected i.p. with either 1 ml of vehicle (0.9% saline), leptin (recombinant rat leptin; PeproTech EC Ltd, London, UK; 50 μg/rat), or CCK-8 [Tocris, Bristol, UK; 15 μg/rat; as described previously (20)]. In a second experiment, 24-h fasted rats (300–320 g body weight) were injected i.p. with 1 ml of vehicle or leptin (50 μg/rat) followed by CCK-8 (15 μg/rat) 15 min later. Doses of leptin in the range 0.1–1 mg/kg have previously been shown to be effective in inducing Fos expression and nuclear pSTAT3 in the VMH and in the arcuate nucleus (18, 21, 22). Leptin has a half-life in the circulation of approximately 9 min in rats (23), and *in vivo* studies of the electrophysiological effects of leptin (in the PVN and on midbrain dopamine neurones) have indicated maximal effects at approximately 20 min after injection (24, 25). Thus, we administered leptin by a physiological (systemic) route, at a dose in the physiological range, and timed the CCK injection to coincide with the expected peak of neuronal responsiveness to systemically applied leptin.

At 90 min after the first injection, rats were killed with an overdose of sodium pentobarbitone (1 ml i.p.) and transcardially perfused with heparinised 0.9% saline, followed by 4% paraformaldehyde in 0.1 m phosphate-buffered saline. The brains were removed, post-fixed overnight, cryoprotected in 30% sucrose, and stored at −20 °C before processing for immunocytochemistry.

### Immunocytochemistry

Coronal brain sections (44 μm) were cut on a freezing microtome. Free-floating sections were immunostained using a polyclonal antibody raised in rabbit against Fos, the N-terminal amino-acids 4–17 of the protein product of human c-*fos* (Ab-2; Calbiochem–Novabiochem, Nottingham, UK) diluted at 1 : 1000 in a pre-incubation phosphate buffer containing 1% normal goat serum. NTS noradrenergic neurones are known to be the source of the afferent pathway to the PVN and SON through which CCK exerts its effects (26). To identify any potential modulation in the neuronal activity of the NTS noradrenergic neurones, some brainstem sections were also double immunostained for Fos and tyrosine hydroxylase (TH; the rate limiting enzyme in noradrenaline synthesis) using a monoclonal anti-TH antibody raised in mouse (Chemicon/Millipore, Watford, UK) at 1 : 1000 in a pre-incubation phosphate buffer containing 1% normal goat serum. To detect pSTAT3 expression, which reflects activation of the leptin receptor, hypothalamic sections were pre-incubated in tris citrate buffer (pH 8.5) at 80 °C, and incubated overnight with a rabbit anti-pSTAT3 (Tyr 705) antibody (Cell Signaling Technology, Danvers, MA, USA) at 1 : 1000 in a phosphate buffer containing 5% normal horse serum. For immunostaining, antibody-antigen complexes were visualised using ABC methods with a Vector stain elite kit (Vector Laboratories, Bucks, UK) with nickel-intensified diaminobenzidine (Ni-DAB; for Fos, black nuclear label) or with DAB only [for TH or pSTAT3, brown cytoplasmic or nuclear label respectively as descrined previously (27)]. Immunoreactivity was analysed in blind-coded sections using a Leica microscope (Leica Microsystems, Wetzlar, Germany). Fos-positive neuronal nuclei were counted in the SON at the level of maximal cross-sectional area, in the PVN (1.80 mm posterior to bregma by reference to (28), in the arcuate nucleus (from 2.30–3.30 mm posterior to bregma) in the dorsomedial hypothalamus (from 2.56–3.14 mm posterior to bregma) and in the VMH (from 2.30–2.80 mm posterior to bregma) as well as in the NTS (from 13.30–13.80 mm posterior to bregma) and in the area postrema (from 13.68–13.80 mm posterior to bregma). In the PVN, magnocellular and parvocellular regions were counted separately by comparing sections with brain atlas sections (28). At the level counted, the parvocellular PVN includes the dorsal cap and ventromedial subdivisions. At least three sections per rat were counted for each area.

### Statistical analysis

Fos and pSTAT3 expressions in different brain areas were compared between groups using a t-test or Mann–Whitney rank sum test where the experimental design involved comparing just two treatments. For the experiment involving more than two groups, one-way anova or anova on ranks were performed followed by pairwise testing using parametric tests (Newman–Keuls) or their nonparametric equivalents (Dunn’s Method) where normality assumptions were violated. P < 0.05 was considered statistically significant.

## Results

### Effects of i.p. CCK or leptin on Fos expression in the hypothalamus and caudal brainstem

**Fig. 1:**
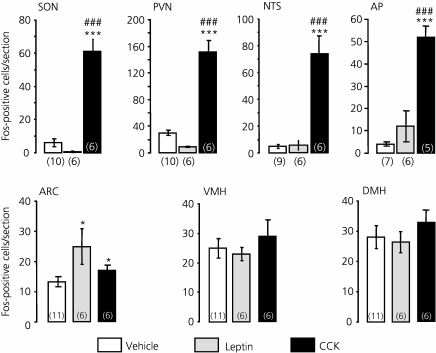
Effects of i.p. leptin or i.p. cholecystokinin (CCK) on Fos expression in the hypothalamus, in the area postrema and in the nucleus tractus solitarii (NTS) of fasted rats. Mean Fos-positive cells/section ± SEM. The number of rats per group is shown in parentheses. *P<0.05 or ***P<0.01 versus vehicle-treated group; ###P<0.01 versus leptin-treated group (one-way anova followed by Student–Newmann–Keuls method or anova on ranks followed by Dunn’s method). AP, Area postrema; SON, supraoptic nucleus; PVN, paraventricular nucleus; ARC, arcuate nucleus; VMH, ventromedial hypothalamus; DMH, dorsomedial hypothalamus.

To establish the effects of CCK or leptin alone on Fos expression in fasted rats, rats without access to food for 24 h were injected i.p. with either vehicle, leptin or CCK. As for fed rats, i.p injections of CCK in fasted rats significantly increased Fos expression in the SON, and in the PVN compared to vehicle-injected rats (Fig. 1; P<0.001) in both magnocellular and parvocellular regions. CCK also induced a modest but significant increase in Fos expression in the arcuate nucleus, although there was no significant effect on Fos expression in the VMH or DMH. CCK significantly increased Fos expression in the area postrema and in the NTS, including in TH-containing neurones; 20% of TH-containing neurones in the NTS expressed Fos in rats treated with CCK compared to only 4% of TH cells in vehicle- treated rats, n = 6 per group, Mann–Whitney rank sum test; P = 0.015; result not shown).

Consistent with previous studies (29), i.p. injections of leptin alone increased Fos expression in the arcuate nucleus of fasted rats, but had no significant effect on Fos expression in the SON, PVN, VMN, DMH, area postrema or in the NTS (Fig. 1). Although leptin apparently inhibited Fos expression in both the SON and in the PVN, these effects were not statistically significant (P = 0.300).

### Effects of i.p. leptin, CCK and leptin + CCK on pSTAT3 expression in fasted rats

**Fig. 2:**
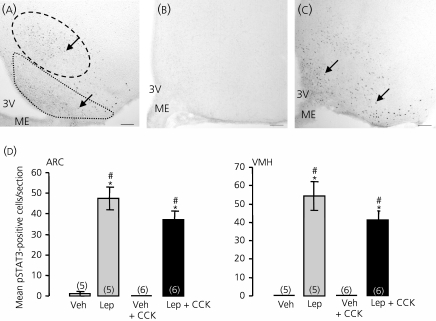
Effects of i.p. leptin, i.p. cholecystokinin (CCK) and i.p. leptin and CCK on pSTAT3 expression in the arcuate nucleus and in the ventromedial hypothalamus of fasted rats. Photomicrographs illustrating single pSTAT3 immunocytochemistry in the arcuate nucleus (dotted line) and in the ventromedial hypothalamus (VMH) (dash line) after leptin (a), vehicle and CCK (b) and leptin and CCK (c) i.p. injections. Black arrow: pSTAT3-positive neurone. Scale bar = 100 μm. 3V, Third ventricle; ME, median eminence. (d) Mean ± SEM pSTAT3-positive cells/section. The number of rats per group is shown in parentheses. *P<0.05 versus vehicle-treated group; #P<0.05 versus vehicle + CCK- treated group (anova on ranks followed by Dunn’s method).

Classically, the actions of leptin in the brain commonly result in phosphorylation of STAT3, which is then translocated to the cell nucleus. To confirm that the dose of leptin used was physiologically active, we therefore measured nuclear pSTAT3 expression in the hypothalamus on selected sections from both experiments. In leptin-injected rats, there was significantly more nuclear pSTAT3 expression in the arcuate nucleus and in the VMH than in vehicle-injected rats (Fig. 2a, d), although we saw no evidence of nuclear pSTAT3 expression in the SON, PVN or DMH (data not shown). CCK alone had no significant effect on pSTAT3 expression in any region of the hypothalamus studied, including the arcuate nucleus and VMH (Fig. 2b, d), and had no effect on responsiveness to leptin because there was no significant difference in the pSTAT3 expression seen in the VMH and in the arcuate nucleus between rats injected with leptin and rats injected with leptin and CCK (Fig. 2c, d).

### Effects of i.p. leptin on CCK-induced Fos expression in fasted rats

**Fig. 3:**
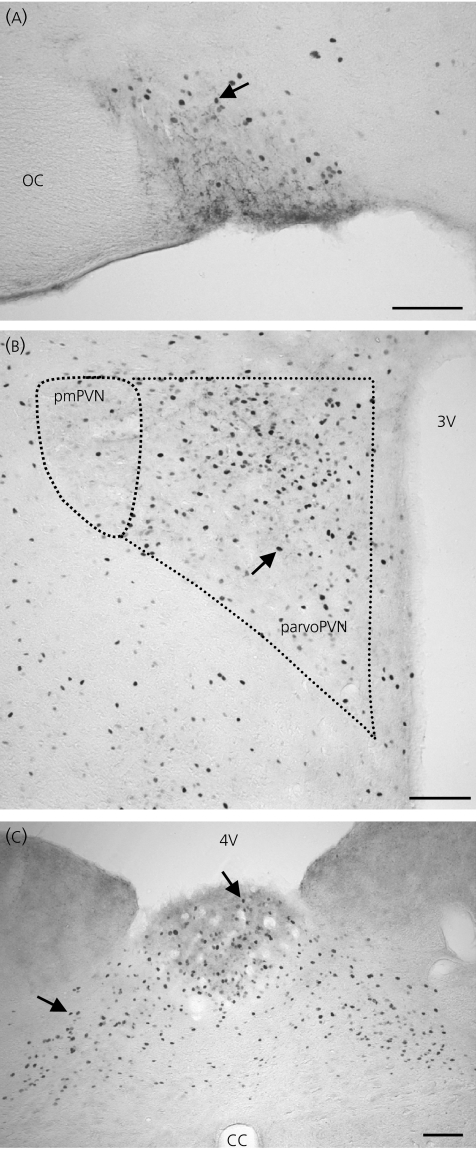
Photomicrograph illustrating Fos immunocytochemistry in the supraoptic nucleus (SON) (a), in the paraventricular nucleus (PVN) (b), in the nucleus tractus solitarii (NTS) and in the area postrema (c) of fasted rats after i.p. leptin and cholecystokinin injections. Black arrow: Fos-positive neurone. Scale bar = 100 μm. pmPVN, Magnocellular divisions of the paraventricular nucleus; parvoPVN, parvocellular subdivisions of the paraventricular nucleus; OC, optic chiasm; 3V, third ventricle; AP, area postrema; CC, central canal; 4V, fourth ventricle.

**Fig. 4:**
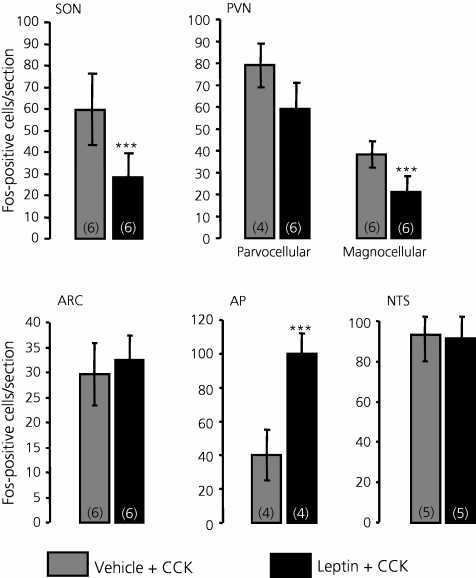
Effects of i.p. leptin and cholecystokinin (CCK) on Fos expression in the supraoptic nucleus (SON), in the parvocellular and magnocellular paraventricular nucleus (PVN), in the arcuate nucleus, in the area postrema and in the nucleus tractus solitarii (NTS) of fasted rats. Mean ± SEM Fos-positive cells/section. The number of rats per group is shown in parentheses. ***P<0.01 versus vehicle + CCK-treated group (*t*-test or Mann–Whitney rank sum test). ARC, Arcuate nucleus; AP, area postrema.

To test the prediction that the responses to CCK in fasted rats would be enhanced by pretreatment with leptin, 24-h fasted rats were treated with either i.p. vehicle or i.p. leptin and then injected with i.p. CCK. Fos expression was then analysed in the hypothalamus and caudal brainstem. There was no significant difference in Fos expression between rats treated with leptin and CCK and rats treated with vehicle and CCK in either the arcuate nucleus or the NTS (Figs 3 and 4) and, within the NTS, the proportion of TH neurones that expressed Fos was also similar in both groups (data not shown). By contrast, Fos expression in both the SON and in the magnocellular PVN was unexpectedly but significantly reduced in rats injected with leptin and CCK compared to rats injected with vehicle and CCK (Figs 3 and 4). Fos expression in the area postrema was significantly increased in rats treated with both leptin and CCK compared to vehicle and CCK (Figs 3 and 4; P < 0.001). There was no significant difference in Fos expression in the parvocellular PVN (counts from dorsal and ventromedial subdivisions combined).

## Discussion

In the present study, we tested the hypothesis that leptin and CCK interact synergistically in signalling to the hypothalamus. In fasted rats, with low circulating levels of leptin, we evaluated CCK responses by quantifying Fos expression in discrete areas of the hypothalamus. We then investigated the effects of pretreating fasted rats with leptin on the measured responses to CCK, using a dose that was effective in increasing pSTAT3 expression in the arcuate nucleus and VMH.

We found that leptin potentiated CCK-induced Fos expression in the area postrema, a circumventricular organ that lies outside the blood–brain barrier, and which is involved in regulation of energy intake. The area postrema contains neurones that are directly excited by circulating CCK (30); thus, the Fos expression in this area that is induced by CCK is therefore likely, in part at least, to reflect a local action, but may also involve indirect actions via connections between the area postrema and the adjacent NTS (31).

Previous studies (32, 33) have reported that i.c.v. administration of leptin potentiates CCK-induced Fos expression in both the NTS and the area postrema. In the present study, we saw that systemically applied leptin potentiated CCK responses only in the area postrema with no effect in the NTS. This suggests that leptin and CCK interact directly at the level of the area postrema, although it is not clear at present whether leptin receptors are also expressed in the area postrema (31). Because no potentiation was seen in the NTS (the source of the major direct projection to the hypothalamus from this region of the caudal brainstem), it is unlikely that leptin potentiation of CCK responses in the area postrema will affect hypothalamic networks, although it might influence efferent regulation of the gastric system (31). The existence of leptin receptors in the NTS remains uncertain because of conflicting observations (12, 34, 35). By contrast to previous studies (32, 33, 36, 37), we saw no effect of leptin on Fos expression in the NTS, suggesting that, at this dose and by this route, leptin has no effect on the NTS; differences between the present results and those of previous studies (32, 33) probably reflect differences in the doses used and the routes of administration.

The present study was focussed on interactions expressed in the response of neurones in the SON and PVN. We used a dose of CCK that is very well characterised in terms of its effects on the electrical activity of magnocellular neurones, its effects on neurohypophysial hormone secretion, and its effects on Fos expression throughout the rat brain (20, 38, 39). We used leptin by a physiological route of administration, at a dose that was adequate to induce pSTAT3 expression in the arcuate nucleus and VMH [confirming previous reports in fed mice (16)]. Previous studies have reported that peripherally-administered leptin also induces Fos expression in the VMH of fed rats (21), but we found no response in fasted rats in the present study.

Unexpectedly, we found that leptin does not potentiate but dampens the effects of CCK on Fos expression in the magnocellular neurones of the SON and PVN. It is likely that this reflects a direct action of leptin on these neurones because it has been reported that they express a high density of leptin receptors (13), and because we saw no similar change in the NTS, the origin of the afferent pathway that mediates the activation of oxytocin neurones by CCK. Because Fos expression is usually interpreted as a marker of neuronal excitation, the natural interpretation is that, in fasted rats, leptin inhibits cell activity in the SON and PVN, suppressing the responsiveness of these neurones to CCK-stimulated pathways. However, Fos expression does not consistently parallel changes in electrical activity (40). In oxytocin neurones of the SON, Fos expression in response to CCK or in response to hypertonic saline injection parallels the increased electrical activity in fed rats (20), although i.c.v. administration of α-MSH induces Fos expression in oxytocin neurones yet reduces their electrical activity (41). In anaesthetised rats, the electrical activity of both oxytocin and vasopressin neurones is modestly but consistently activated in response to injections of leptin (42). Thus, although the direct actions of leptin on magnocellular neurones appear to result in a suppression of Fos expression, they do not result in a simultaneous suppression of electrical activity.

We saw no induction of pSTAT3 expression in the SON. Consistent with this, although i.c.v. leptin strongly induces nuclear STAT3 immunoreactivity in the arcuate nucleus, VMH and DMH, it does not do so in the SON (43), whereas nuclear STAT3 immunoreactivity in this nucleus can be induced by interleukin-6 (44). It appears that, although supraoptic neurones express leptin receptors in abundance, systematically applied leptin does not reach the SON or the receptors in the SON do not signal via STAT3. It is possible, however, that they may signal via nuclear translocation of STAT5; in a study by Mutze *et al.* (45), systemic administration of a high dose of leptin induced intense nuclear STAT5 staining in the SON as well as the arcuate nucleus, but not in the VMH or DMH.

In summary, leptin signals in the brain involve distinct signalling pathways in different neuronal populations. In oxytocin neurones, leptin receptors may signal via STAT5 rather than via STAT3. Oxytocin mRNA expression is reduced during fasting and this reduction can be reversed by i.p. injection of leptin (19); hence, we propose that the actions of leptin may involve an up-regulation of oxytocin mRNA expression that contributes to a long-term enhancement of oxytocin secretion in response to afferent stimuli. However, these signalling pathways that are activated by leptin appear to suppress rather than potentiate the expression of Fos protein, indicating that this transcription factor is not involved in leptin-induced up-regulation of oxytocin expression.
